# Dietary Practice and Associated Factors among Pregnant Women in Misha Woreda, South Ethiopia: A Community-Based Cross-Sectional Study

**DOI:** 10.1155/2020/5091318

**Published:** 2020-09-25

**Authors:** Lonsako Abute, Abera Beyamo, Belay Erchafo, Tegegn Tadesse, Dawit Sulamo, Tagesse sadoro

**Affiliations:** Public Health Department, College of Medicine and Health Sciences, Wachemo University, Hossana, Ethiopia

## Abstract

**Background:**

Proper food and good nutrition are essential for survival, physical growth, mental development, performance and productivity, and health and well-being. Pregnancy is a critical phase in a woman's life. The aim of this study is to assess the dietary practice and associated factors among pregnant women in Misha woreda, south Ethiopia.

**Methods:**

A cross-sectional study was conducted in Misha woreda, South Ethiopia, on pregnant women. Data were collected by using a structured interviewer-administered questionnaire. The data were entered in EpiData V-3.1 and analyzed using SPSS version 21. Binary logistic regression analysis was also employed to examine the association between dependent and independent variables. A *P* value of <0.05 was considered as the cutoff point to declare statistical significance.

**Result:**

Out of 618 pregnant women, almost all of them 618 interviewed with the response rate of 100%. The mean age of pregnant women was 27.31 years (±5.622). From total study participants, 54.1% of the respondents were followers of protestant religion and 80.2% of husband occupation were farmers and 78.7% pregnant women occupation were house wives. From the total participants, 43.6% had illness on the current pregnancy. Almost two third 66.2% of the pregnant women travel ≥ 1 hr to reach HF. Majority of the participants (62%) had moderate knowledge about dietary practice in pregnancy, and 29.5% practiced good dietary practice. Educational status (AOR = 4.07 [2.13, 9.18]), occupation (AOR = 5.32 [1.08, 13.95]), dietary knowledge (AOR = 7.2 [3.9, 17.09]), and food craving (AOR = 2.07 [1.41, 5.5]) were variables having a significant association with dietary practice.

**Conclusion:**

The prevalence of good dietary practice among pregnant women in Misha district was low when compared to other studies. According to the study result, educational status, occupation, dietary knowledge, and food craving were factors that affect dietary practice.

## 1. Background

Both developed and developing countries have the problem of nutritional problem; it may be under or over nutrition, but pregnent mothers are more vulnerable for it [1]. Nutritional status of pregnant mother determine health and well-being of the mother and her child. This mother requires more energy and nutrients to meet the need of the growing fetus and maternal tissues [2]. Therefore, practicing dietary balance ensures sufficient energy intake for growth of the fetus [3]. In Ethiopia, nutritional disorders are among the main causes of maternal morbidity and mortality. The major nutritional disorders are protein-energy malnutrition and micronutrient deficiencies in this country [4]. Twenty-two percent of women are undernourished with a body mass index (BMI) less than 18.5 [5].

Pregnant woman are in a particular risk for nutritional deficiencies if they were adolescent, underweight, smokers, alcohol/drugs users and have chronic nutritional problems or chronic illnesses [6]. Poor nutrition of women with lack of care contributes to death in pregnant mother [7]. The mother's death also compromises health and survival of the infant and children. The effects of maternal under nutrition peak during pregnancy and within the first two years of child. Furthermore, effects on brain development, intelligence, educability, and productivity are mainly irreversible [8].

The adequate maternal nutrition is one of the best ways to ensure maternal and fetal well-being. Therefore, good dietary practice of pregnant mother reduces the risk of chronic diseases. The consistent evidence about the pregnant mothers' dietary practice is lacking in the study area. So, this study aimed to assess the dietary practice and associated factors among pregnant women in Misha woreda, Southern Ethiopia.

## 2. Methods

### 2.1. Study Design Period and Area

A community-based cross-sectional study was conducted in pregnant women in Misha woreda, from April 1 to May 30, 2019. The woreda contains 23 kebeles (2 urban and 21 rural kebeles) with a total population of 103,000 with 49.47% of male and 50.53% of female. In the woreda, there are 3,564 expected numbers of pregnant women. As to health infrastructures, there are three functional health centers.

### 2.2. Study Population and Sampling

All pregnant women who live in Misha woreda were considered as the source population, and sampled pregnant women in the randomly selected kebeles were taken as the study population.

Sample size was calculated by using the single population proportion formula based on the following parameters: 95% confidence level (1.96), margin of error (0.05) and 27%, the prevalence of good dietary practice as during pregnancy from Gondar (13) and design effect 2, and 10% none response. The calculated sample size was 618 pregnant mothers in Misha Woreda, Hadiya Zone, Southern Ethiopia. To access study participants, first of all, the kebeles were stratified into urban and rural. There are 21 rural and 2 urban kebeles in the woreda. So that, 1 urban and 7 rural with a total of 8 kebeles were taken randomly. After doing preliminary assessment to identify those pregnant mothers in the selected kebeles, sample seize was distributed proportional to population size for each kebeles. Finally, the systematic sampling technique was used to select 618 pregnant women by preparing the sampling frame.

### 2.3. Data Collection Instrument and Data Collectors

A quantitative method of data collection was employed for assessment of dietary practice among pregnant mothers in Misha district, Hadiya Zone, Southern Ethiopia, by using a face-to-face interviewer-administered questionnaire. The tool has six sections such as sociodemographic characteristics of the respondents (9 questions), obstetric and related characteristics (6 questions), health services and household-related characteristics (5 questions), dietary knowledge of pregnant mothers (10 questions), meal pattern of pregnant mothers (11questions), and dietary practice of pregnant mothers (13 questions). Dietary practice was determined using questions related to dietary practices of pregnant mothers. The questionnaire of practice on food and nutrition was used in this study to measure dietary practices. Scores of dietary practices were obtained by summation of each group of questions. Each question was given one mark if the answers are correct, favorable, or healthy for dietary practice. Zero score was given if the responses are wrong, unfavorable, or unhealthy for dietary practice questions. The score of the respondents was taken, and respondents were classified as having good or poor dietary practices by taking their responses ≥75% and below 75%, respectively.

### 2.4. Data Quality Control

For administering the structured questionnaire, four BSc and eight diploma nurse professionals were employed a supervisor and data collector, respectively, in the woreda. The questionnaire was prepared in English and, then, translated into the local language and translated back into English language by another language expert to check consistency. Two days of training was given for data collectors and supervisors on the objective, relevance of the study, confidentiality of information, respondent's right, time of data collection from the kebeles, and submission on due time. The pretest was conducted on 5% of the actual sample size in Gibe woreda pregnant women. The collected data were checked for completeness and consistency in daily basis.

### 2.5. Data Processing and Analysis

Data were entered into EpiData version 3.1and exported to SPSS 20.0 statistical software to edit, clean for inconsistencies and missing values, and finally, to analyze. Descriptive analysis was carried out for each of the variables to check frequency, distribution, and missing value. Bivariate logistic regression analysis was employed to check crude association between dietary practice and independent variables. Variables with a *p* value <0.25 on bivariate logistic regression analysis were entered into multivariable logistic regression to identify the factors that affect dietary practice. Odds ratio and the corresponding 95% confidence intervals were used to quantify the degrees of association between the independent variable and the outcome variable. Results with a *p* value <0.05 were considered as being statistically significant, and the rest was refuted. Multicollinearity among independently associated variables was checked by a multicollinearity diagnostic test VIF in linear regression, and none was collinear.

#### 2.5.1. Dietary Practices

Seven dietary practice questions were designed to assess practices of mothers on nutrition during pregnancy. The result was obtained by summation of each group of questions. Each question was given one mark if the answer is correct, favorable, or healthy for dietary practice. Zero score was given if the responses are wrong, unfavorable, or unhealthy for dietary practice questions. The score of the respondents was taken, and respondents were classified as good dietary practice if the score >75% and poor dietary practices if below 75% [9, 10].

#### 2.5.2. Knowledge of Dietary Practice

Eight dietary knowledge question was asked, and each item was given a score of 1 (correct) or 0 (incorrect), and the sums were used as the knowledge scores. Finally, it was classified as good knowledgeable if the score was >70% and poor knowledgeable if below 70% [9, 10].

#### 2.5.3. Meal Pattern

Avoidance or skipping from the habitual meal relating with the pregnancy outcome.

## 3. Results

### 3.1. Sociodemographic Characteristics

Out of 618 pregnant women, almost all of them were interviewed for this study and yielding their response rate of 100%. The mean age of pregnant women was 27.31 years (±5.622). From total study participants, 334 (54.1%) of the respondents were followers of protestant, and 225 (36.4%) were orthodox. Among the study subjects, the occupation of husbands of 495 (80.2) was farming, and 486 (78.7%) pregnant women were house wives. Majority of the respondents' (231 (37.3)) educational status was can read and write. Two hundred and sixty (42) of the respondents have the family size of 3-4. Regarding average monthly income of the family, more than half (184 (54.4%)) got less than 2000 birr per month, and 336 (54.4%) of them got <2000 birr per month (Table 1).

### 3.2. Obstetric and Health Service-Related Factors of the Pregnant Women

From a total of 618 participants, 284 (46%) pregnant mothers were in the second trimester of the pregnancy and 429 (69.3%) of them had 3–5 pregnancies before the current pregnancy and 309 (50%) had 2–5 years gap duration between previous and current pregnancy. From the total participants, 269 (43.6%) had illness on the current pregnancy, and headache and blurred vision were the leading illnesses (186 (69%)). Almost two-third (409 (66.2%)) of the pregnant women travel ≥1 hr to reach HF. Only 28.2% of the participants got support from their husband during their pregnancy nutritionally, and the majority (112 (64.6%)) were supported by reminding to consume foods or supplements (Table 2).

### 3.3. Dietary Knowledge of the Pregnant Women

It is revealed that the majority of the participants (383 (62.0%)) had moderate knowledge about dietary practice in pregnancy (Table 3).

### 3.4. Meal Pattern of Pregnant Mothers

From a total of 618 respondents, 36.9% pregnant women consume additional meal and 83.3% of those have one extra meal within a day. From those who did not consume additional meal, poor economy (39.6%) and considered as adequate (31.5%) were the main reasons. One hundred and twenty-nine (20.9%) of the respondents have the history of skipping meal, and personal dislike (36.8%) and fear of obesity (35.1%) were the major ones. From the total participants, 33% practiced food avoiding, and majority (52.1%) of them avoid due to cultural reasons such as it will make baby big and labor difficulty (33.6%), will be plastered on the fetal head (37.9%), and evil eye (28.5%). A total of 256 (41.2%) of the participants have food desire strongly (craving), and food odor (47.4%) accounts the major reason to crave food items. Almost one-third of them did not get the craved food, and the main reason for not getting the craved food was nonavailablility (71.3%) (Table 4).

### 3.5. Dietary Practices of Pregnant Mothers during Pregnancy

**Figure 1:**
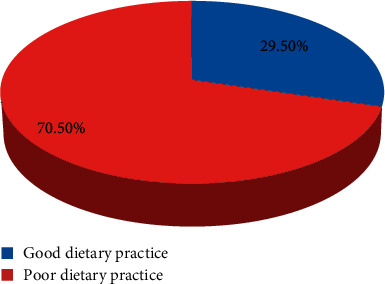
Dietary practices of pregnant mothers during pregnancy in Misha woreda, 2019.

From the total of 618 participants, 181 (29.5%) practiced good dietary practice while 437 (70.5%) had poor dietary practice (Figure 1).

To identify factors associated with dietary practices, both binary and multivariate logistic regression models were used. Accordingly, factors that were associated with dietary practices of a pregnant mother under binary logistic regression were age of pregnant women, educational status of pregnant women, monthly income, occupation of pregnant women, husband support, gap between pregnancy, dietary knowledge, additional meal, meal skipping, and food craving which were significantly associated with dietary practice of pregnant women at a *p* value 0.25 (Table 5).

### 3.6. Factors That Affect Dietary Practice among Pregnant Mothers

The variables that showed a significant association with dietary practice during pregnancy were adjusted for their confounders using the multivariate logistic regression educational status, occupation, monthly income, dietary knowledge, additional meal, and food craving that became independent predictors for dietary practices. Those literate pregnant women were four times (AOR = 4.07 [2.13, 9.18]) more likely good dietary practice than illiterate. Those who had an estimated family average monthly income of ≥4000 birr were 5 times (AOR = 5.32 [1.08, 13.95]) more likely to have good dietary practice than those who had an estimated family monthly income less than 2000.00 birr (AOR = 5.32[1.08, 13.95]). Pregnant mothers of government employers were seven times (AOR 7.2 [3.9, 17.09]) more likely to have good dietary practice than house wives. Also, those pregnant women with high dietary knowledge were eight times (AOR = 8.53 [2.19, 21.05]) more likely to have good dietary practice than mothers of poor dietary knowledge. Pregnant mothers who take additional meal were four times (AOR = 4.7 [1.6, 10.3]) more likely to have good dietary practice than mothers with no additional meal, and those mothers with no food craving were two times (AOR = 2.07[1.41, 5.5]) more likely to have good dietary practice than mothers with food craving (Table 6).

## 4. Discussion

In this study, only 29.5% of the pregnant mothers were found to have good dietary practice during their pregnancy. The finding of this study is higher than that of a study done in the West Gojjam Zone (19.9%) [9]. The study finding is lower than the study findings of Bahir Dar town (39.3%) [11], Dessei town (45.2) [12], Jille Tumuga district (31.4%) [13], and Gonder town (40.10%) [10]. The reason for this discrepancy might be due to differences in sociodemographic characteristics. Most of the studies have been conducted at towns which have different living conditions.

This study showed the educational status as significant factor with dietary practice. Literate mothers have good dietary practice when compared to illiterates. This finding was supported with another study conducted at America [14] and Gondar town [10]. The reason might be the demographic factors and accessibility difference among the study populations.

This study indicates that the mother's occupation was a significant factor of dietary practice of a pregnant mother. Mothers engaged in government work were seven times more likely to practice good dietary practice than house wives (AOR = 7.2, 3.9, 17.09). This finding is supported by a previous study conducted in Kenya [15] and in Addis Ababa [16]. The reason might be that those mothers engaged in government work may be more accessible to get more information about the diet during pregnancy and they may adhere to practice more than house wives.

Moreover, in this study, women's dietary knowledge had shown a positive relationship with dietary practice of mothers during pregnancy (AOR = 8.5, 2.19–21.05). This finding is supported by the study conducted in West Gojjam [9], Kenya [15], and Addis Ababa [16]. The reason behind the similarity might be due to the fact that when the women is exposed to dietary information, they may be informed of the consequences of undernutrition on their children, as well as on themselves, and they will more enforced to practice adequate diet.

Presence of food craving has a negative association with good dietary practice. Those mothers with no food craving were two times more likely to practice good dietary practice than those with food craving (AOR = 2.07, 1.41–5.5).

## 5. Conclusions

The prevalence of good dietary practice among pregnant women in Misha district was low when compared to other studies. According to the study result, being literate, being government employee, and having good dietary knowledge were facilitators of good dietary practice whereas food craving was an inhibitor of good dietary practice.

### 5.1. Recommendation

Based on the findings, the following recommendations were forwarded for identified gaps: Misha district health office, health extension worker, and health workers due attention to maximize the prevalence of good dietary practice by awareness creation of pregnant mothers. The Department of Public Health, Wachemo University, College of Medicine and Health Science, should also takes responsibility for further assessments on dietary practice to identify additional factors affecting dietary practices of pregnant mothers. The Wachemo University Research and Community Service Directorate should prepare interventional training for pregnant mothers towards dietary practice on identified gaps.

## Figures and Tables

**Table 1:** Sociodemographic characteristics of pregnant mothers in Misha woreda, 2019.

Explanatory variables	Category	Frequency	Percent
Age	18–26	296	47.9
	27–35	258	41.7
	≥36	64	10.4

Religion	Protestant	334	54.1
	Orthodox	225	36.4
	Muslim	46	7.4
	Catholic	13	2.1

Marital status	Married	583	94.4
	Widowed	25	4.0
	Divorced	10	1.6

Husband educational status	No formal education	143	23.1
	Can read and write	230	37.2
	Primary	148	23.9
	Secondary and above	97	15.7

Husband occupation	Employed	42	6.8
	Merchant	48	7.7
	Daily labourer	33	5.3
	Farmer	495	80.2

Maternal education	No formal education	184	29.8
	Can read and write	231	37.3
	Primary	177	28.7
	Secondary and above	26	4.2

Maternal occupation	House wife	486	78.7
	Government employed	23	3.8
	Merchant	48	7.7
	Daily laborer	61	9.8

Family size of respondents	≤2	137	22.2
	3-4	260	42.0
	≥5	221	35.8

Average monthly income (Ethiopian birr)	<2000.00	336	54.4
	2000.00–4000.00	227	36.7
	>4000.00	55	8.9

**Table 2:** Obstetric and health service-related factors of the pregnant women in Misha woreda, 2019.

Explanatory variable	Category	Frequency	Percent
Trimester of the pregnancy	First	93	15.0
	Second	284	46.0
	Third	241	39.0

Number of pregnancies before the current pregnancy	≤2	165	26.8
	3-5	429	69.3
	5+	24	3.9

Gap duration between the past and current pregnancy	≤2 years	263	42.5
	3–5 years	309	50.0
	5+ years	46	7.5

History of illness on current pregnancy	Yes	269	43.6
	No	349	56.4

Kind of illness	Headache and blurred vision	186	69.0
	Lower abdominal pain	35	13.0
	Vomiting and epigastric pain	86	32.0

Time of initiation of ANC visits	<3 months of pregnancy	130	21.0
	3–6 months	414	67.0
	>6 months	74	12.0

Number of ANC visits	≤4 visits	569	92.0
	*>*4 visits	49	8.0

Time that takes to reach HF	<1 hr	209	33.8
	≥1 hr	409	66.2

Husband support	Yes	174	28.2
	No	444	71.8

The way he supports you	By purchasing diverse foods/supplements	62	35.4
	By reminding me to consume these foods or supplements.	112	64.6

**Table 3:** Dietary knowledge of the pregnant women in Misha woreda, 2019.

Knowledge category	Frequency	Percent
High	173	28.0%
Moderate	383	62.0%
Low	62	10.0%

**Table 4:** Meal pattern of pregnant mothers in Misha woreda, 2019.

Variables	Category	Frequency	Percent
Additional meal consumed	Yes	228	36.9
	No	390	63.1

Number of extra meals within a day	Once	190	83.3
	Twice	38	16.7

Reason of not having an additional meal	Lack of information	113	28.9
	Poor economy	154	39.6
	Considered as adequate	123	31.5

Skipping meal	Yes	129	20.9
	No	489	79.1

Reason of meal skipping	Personal dislike	47	36.8
	Fear of obesity	45	35.1
	Poor economy	37	28.1

Fasting habits during pregnancy	Yes	48	7.8
	No	570	92.2

Food avoided during pregnancy	Yes	204	33.0
	No	414	67.0

Reason of food avoidance	Personal dislike/aversion	98	47.9
	Not allowed to pregnant women/cultural taboo	106	52.1

Reason of cultural beliefs/taboo	Will make baby big and labour difficulty	36	33.6
	Will be plastered on the fetal head	40	37.9
	Evil eye	30	28.5

Any food desired strongly (craving)	Yes	256	41.2
	No	363	58.8

Reason to crave for these food items	Colour of food	52	20.4
	Food odor	121	47.4
	I do not know the reason	83	32.2

Did you get the food you crave	Yes	170	66.4
	No	86	33.6

The reason of not getting craved food	Not affordable	25	28.7
	Not available	61	71.3

**Table 5:** Binary logistic regression with its COR for variables predicting dietary practice among pregnant mothers, Misha woreda, 2020.

Variables	Dietary practice		COR [95% CI]	*p* value
	Good	Poor		
Age				
18–26	56 (9.10%)	240 (38.83%)	0.34 [1.01, 13.05]	0.03
27–35	97 (15.70%)	161 (26.05%)	0.95 [0.23, 6.02]	0.23
≥36	28 (4.53%)	44 (7.12%)	1	

Marital status				
Married	150 (24.27%)	413 (66.83%)	0.21 [0.05, 2.03]	0.62
Divorced	9 (1.46%)	11 (1.78%)	0.48 [0.23, 6.72]	0.81
Widowed	22 (3.56%)	13 (2.10%)	1	

Occupation				
Merchant	20 (3.24%)	28 (4.53%)	0.77 [0.20, 2.97]	0.12
Daily labourer	31 (5.02%)	30 (4.85%)	0.46 [0.17, 1.21]	0.21
Government employee	37 (5.99%)	16 (2.59%)	0.40 [0.15, 1.02]	0.002
House wife	93 (15.05%)	363 (58.74%)	1	

Education				
Read and write	41 (6.63%)	110 (17.80%)	1.26 [0.18, 2.39]	0.22
1–8	42 (6.80%)	135 (21.84%)	1.05 [0.24, 4.39]	0.12
Secondary and above	56 (9.065%)	50 (8.10%)	3.40 [0.52, 8.91]	0.04
Cannot read and write	42 (6.80%)	142 (22.98%)	1	

Income				
<2000	77 (12.46%)	259 (41.91%)	0.20 [0.28, 1.74]	0.15
2000–4000	69 (11.17%)	158 (25.57%)	0.25 [0.20, 1.18]	0.023
>4000	35 (5.66%)	20 (3.24%)	1	

Husband support				
Yes	76 (12.23%)	98 (15.86%)	2.60 [0.46,8.12]	0.14
No	105 (16.99%)	339 (54.85%)	1	

Gap between pregnancy				
≤2 yr	81 (13.11%)	162 (26.21%)	1.52 [1.41,5.02]	0.47
3–5 yr	59 (9.55%)	150 (24.27%)	1.20 [1.28, 4.91]	0.11
>5 yr	41 (6.63%)	125 (20.23%)	1	

Dietary knowledge				
Good	128 (20.71%)	230 (37.22%)	2.17 [2.28,13.02]	0.003
Poor	53 (8.58%)	207 (33.50%)	1	

Additional meal				
Yes	107 (17.31%)	121 (19.58%)	3.80 [1.50,5.86]	0.04
No	74 (11.97%)	316 (50.97%)	1	

Meal skipping				
Yes	31 (5.02%)	98 (15.86%)	0.71 [1.08,11.67]	0.13
No	150 (24.27%)	339 (54.85%)	1	

Food craving				
Yes	91 (14.72%)	113 (18.28%)	2.90 [0.52, 8.91]	0.09
No	90 (14.56%)	324 (52.43%)	1	

Time of initiation ANC visit				
<3 months of pregnancy	69 (11.17%)	165 (26.70%)	0.73 [0.36, 5.85]	0.87
3–6 months of pregnancy	85 (13.75%)	225 (36.41%)	0.66 [0.53, 1.89]	0.98
>6 months of pregnancy	27 (4.37%)	47 (7.61%)	1	

Time that takes to reach health facility				
<1 hour	99 (16.02%)	110 (17.80%)	3.60 [1.28, 4.91]	0.65
≥1 hour	82 (13.27%)	327 (52.91%)	1	

Additional meal				
Yes	112 (18.12%)	116 (18.77%)	4.50 [0.48, 1.94]	0.86
No	69 (11.17%)	321 (51.94%)	1	

**Table 6:** Variables predicting dietary practice among pregnant mothers in logistic regression.

Variables	Dietary practice		COR [95% CI]	AOR (95% CI)
	Good	Poor		
Age				
18–26	80 (12.94%)	211 (34.14%)	0.35 [1.0, 33.5]	2.07 [0.19, 7.9)]
≥27	170 (27.51%)	157 (25.40%)	1	

Education				
Literate	166 (26.86%)	160 (25.89%)	7.22 [1.02,7.6]	4.07 [2.13, 9.18]^*∗*^
Illiterate	31 (5.02%)	261 (42.23%)	1	

Monthly income				
≥4000	160 (25.89%)	176 (28.48%)	0.25 [1.4,6.3]	5.32 [0.08, 13.95]
<4000	221 (35.76%)	61 (9.87%)	1	

Occupation				
Government employed	97 (15.70%)	15 (2.43%)	14.82 [2.6,8.9]	7.2 [3.9, 17.09]^*∗∗*^
Housewife	154 (24.92%)	353 (57.12%)	1	

Husband support				
Yes	76 (12.23%)	98 (15.86%)	5.61 [0.46,8.2]	4.03 [2.01, 8.65]
No	105 (16.99%)	339 (54.85%)	1	

Gap between pregnancy				
≤2 years	74 (11.97%)	189 (30.58%)	0.74 [1.4,5.02]	3.03 [0.61, 7.15]
>2 years	107 (17.31%)	248 (40.13%)	1	

Dietary knowledge				
High	128 (20.71%)	230 (37.22%)	6.90 [2.8,13.2]	8.5 [2.19, 21.05]^*∗∗*^
Low	53 (8.58%)	207 (33.50%)	1	

Additional meal				
Yes	112 (18.12%)	116 (18.77%)	6.71 [4.2,14.2]	5.03 [0.64, 11.3]^*∗*^
No	69 (11.17%)	321 (51.94%)	1	

Skipping meal				
Yes	31 (5.02%)	98 (15.86%)	1.20 [1.2,4.9]	2.3 [0.92, 6.84]
No	150 (24.27%)	339 (54.85%)	1	

Food craving				
Yes	91 (14.72%)	113 (18.28%)	1	
No	90 (14.56%)	324 (52.43%)	1.25 [0.4,6.2]	2.07 [1.41, 5.5]^*∗*^

*∗∗*=*p* value <0.001, *∗*=*p* value <0.05.

## Data Availability

The data used to support the findings of this study are available from the corresponding author upon request.

## Abbreviation

AOR: Adjusted odds ratio
BMI: Body mass index
COR: Crude odds ratio
SPSS: Statistical package for social science.

## References

[1] Shekar M., Heaver R., Lee Y.-K. (2006). *Repositioning Nutrition as Central to Development: A Strategy for Large Scale Action*.

[2] Barker D. J. P., Godfrey K. M., Gluckman P. D., Harding J. E., Owens J. A., Robinson J. S. (1993). Fetal nutrition and cardiovascular disease in adult life. *The Lancet*.

[3] Subarnalata S., Panda B. (2006). A study of nutritional status of pregnant women of some villages in Balasore district, Orissa. *Journal of Human Ecology*.

[4] Federal Democratic Republic of Ethiopia MoH (2010). *Health Sector Development Programme IV 2010/11 – 2014/15*.

[5] Central Statistical Agency and ORC Macro (2016). *Central Statistical Agency [Ethiopia] and ICF International: Ethiopia Demographic and Health Survey*.

[6] Edris M. M., Tekle H., Fitaw Y., Gelaw B., Engedaw T. A. D. (2005). *Maternal Nutrition*.

[7] Abdella A. (2010). Maternal mortality trend in Ethiopia. *Ethiopian Journal of Health Development*.

[8] Infant I. Y. CN., Young (2011). *Child Nutrition Project; Literature Review Prepared for the Message and Materials Development Workshop Produced through Support provided*.

[9] Demilew Y. M., Alene G. D., Belachew T. (2020). Dietary practices and associated factors among pregnant women in West Gojjam Zone, Northwest Ethiopia. *BMC Pregnancy and Childbirth*.

[10] Alemayehu M. S., Tesema E. M. (2015). Dietary practice and associated factors among pregnant women in gondar town north west, Ethiopia. *International Journal of Nutrition and Food Sciences*.

[11] Nana A., Zema T. (2018). Dietary practices and associated factors during pregnancy in northwestern Ethiopia. *BMC Pregnancy and Childbirth*.

[12] Diddana T. Z. (2019). Factors associated with dietary practice and nutritional status of pregnant women in Dessie town, northeastern Ethiopia: a community-based cross-sectional study. *BMC Pregnancy and Childbirth*.

[13] Aliwo S., Fentie M., Awoke T., Gizaw Z. (2019). Dietary diversity practice and associated factors among pregnant women in North East Ethiopia. *BMC Res Notes*.

[14] Shehab L. (2012). Nutritional awareness of women during pregnancy. *Journal of American Science*.

[15] Wahome E. M., Makau W. K., Kiboi W. K. (2018). Predictors of dietary practices and nutritional status among diabetic type II patients in Kiambu County, Kenya. *International Journal Of Community Medicine And Public Health*.

[16] Zelalem T., Erdaw A., Tachbele E. (2018). Nutritional knowledge, attitude and practices among pregnant women who attend antenatal care at public hospitals of Addis Ababa, Ethiopia. *International Journal of Nursing and Midwifery*.

